# Construction and Application of Polygenic Risk Scores in Autoimmune Diseases

**DOI:** 10.3389/fimmu.2022.889296

**Published:** 2022-06-27

**Authors:** Chachrit Khunsriraksakul, Havell Markus, Nancy J. Olsen, Laura Carrel, Bibo Jiang, Dajiang J. Liu

**Affiliations:** ^1^ Graduate Program in Bioinformatics and Genomics, Pennsylvania State University College of Medicine, Hershey, PA, United States; ^2^ Institute for Personalized Medicine, Pennsylvania State University College of Medicine, Hershey, PA, United States; ^3^ Department of Medicine, Division of Rheumatology, Pennsylvania State University College of Medicine, Hershey, PA, United States; ^4^ Department of Biochemistry and Molecular Biology, Pennsylvania State University College of Medicine, Hershey, PA, United States; ^5^ Department of Public Health Sciences, Pennsylvania State University College of Medicine, Hershey, PA, United States

**Keywords:** autoimmune diseases, genome wide association studies (GWAS), multi-ancestry genetic study, polygenic risk score (PRS), electronic health record (EHR)

## Abstract

Genome-wide association studies (GWAS) have identified hundreds of genetic variants associated with autoimmune diseases and provided unique mechanistic insights and informed novel treatments. These individual genetic variants on their own typically confer a small effect of disease risk with limited predictive power; however, when aggregated (e.g., *via* polygenic risk score method), they could provide meaningful risk predictions for a myriad of diseases. In this review, we describe the recent advances in GWAS for autoimmune diseases and the practical application of this knowledge to predict an individual’s susceptibility/severity for autoimmune diseases such as systemic lupus erythematosus (SLE) *via* the polygenic risk score method. We provide an overview of methods for deriving different polygenic risk scores and discuss the strategies to integrate additional information from correlated traits and diverse ancestries. We further advocate for the need to integrate clinical features (e.g., anti-nuclear antibody status) with genetic profiling to better identify patients at high risk of disease susceptibility/severity even before clinical signs or symptoms develop. We conclude by discussing future challenges and opportunities of applying polygenic risk score methods in clinical care.

## Introduction

There are nearly 100 autoimmune diseases, many of which are rare with prevalence of less than 5 per 100,000 individuals (1, 2). Yet, the prevalence of autoimmune diseases is increasing in recent years. The National Institutes of Health estimates that 14.7-23.5 million people (around 4-7% of the population) are affected in the United States overall (3).

Autoimmune diseases arise from a combination of genetic predispositions and environmental factors that result in the loss of self-tolerance and may cause the immune system to mount a response against the body’s own healthy cells and tissues (4). Genetic effects can alter both the innate and adaptive immune systems (5). Likewise, altered immune responses can be triggered by environmental factors like microbial antigens or environmental toxins, although triggers in many of these disorders, remain unclear. This often leads to the production of autoantibodies and activation of cell-mediated autoimmunity. Some autoimmune diseases target specific cell types (e.g., pancreatic ß-cells in type-1 diabetes or thyroid-stimulating hormone (TSH) receptor in Hashimoto thyroiditis), while others can target a common antigen present in a wide range of cell types (e.g., nuclear antigens in systemic lupus erythematous or systemic sclerosis) (6).

The clinical presentation and severity of most autoimmune diseases are heterogenous due to their complex etiology (7). Moreover, symptoms of different disorders can overlap. As a result, autoimmune disease diagnosis remains challenging. Misdiagnoses of autoimmune diseases are common (8–10) and a correct diagnosis can take several years and multiple physician visits (e.g., rheumatology, endocrinology, hematology, etc.). Delayed diagnoses and treatment can allow disease to progress to advanced stages, affecting multiple organ systems, and even leading to fatality. As a result, early diagnosis and proper treatment management of autoimmune diseases is a clinical necessity.

In this review, we discuss the current states of genome wide association studies for a number of autoimmune diseases and how we can leverage those results to develop polygenic risk scores (PRS) for disease risk prediction based on one’s genetic information. We discuss various methods and strategies used to derive PRS models. Finally, in the era of precision using electronic health records, we discuss the clinical utility of combining conventional lab tests with genetic data to improve risk prediction.

## GWAS of Autoimmune Diseases Reveals Genetic Architecture

Genome wide association studies (GWAS) have significantly changed our understanding of the genetic landscape underpinning autoimmune diseases. In this review, we look into 16 autoimmune diseases or traits: ankylosing spondylitis (AS), celiac disease (CEL), Crohn’s disease (CD), Grave’s disease (GD), Hashimoto thyroiditis (HT), multiple sclerosis (MS), primary biliary cirrhosis (PBC), psoriasis vulgaris (PSO), psoriatic arthritis (PSOAR), rheumatoid arthritis (RA), Sjögren’s syndrome (SS), systemic lupus erythematous (SLE), systemic sclerosis (SSC), type 1 diabetes (T1D), ulcerative colitis (UC), and vitiligo (VIT). At the time of this review, there are 179 published GWAS studies that have identified over 350 loci across these 17 autoimmune traits (11).

**Figure 1:**
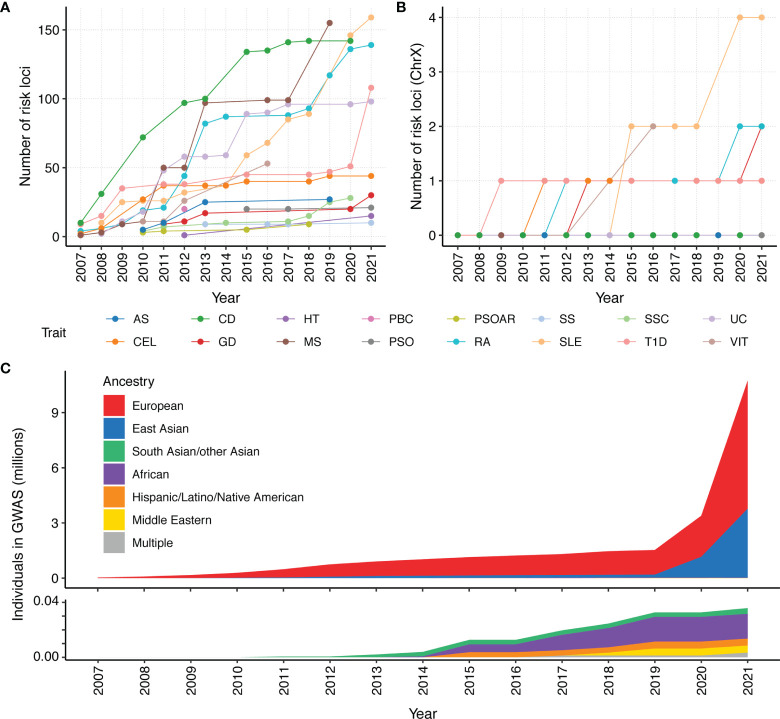
Number of risk loci identified by GWAS for 16 autoimmune traits and ancestry composition per year since 2007. We count the cumulative number of reported loci in GWAS catalog. Each locus is defined as a 1 million basepair window surrounding a genome-wide association signal (p < 5×10^-8^). All significant variants within a 1 million basepair window are attributed to a single locus. The cumulative number of unique loci that were identified in a year were calculated for the **(A)** whole genome and **(B)** chromosome X. Given that the X chromosome represents approximately 5% of the genome, the paucity of X GWAS loci for most autoimmune disorders makes it clear that the X chromosome is understudied. **(C)** Cumulative assessment of GWAS participants by ancestry over time, according to GWAS catalog. A majority of current GWAS studies are from European ancestry. As people of European ancestry only account for 16% of the population, the non-European population remain under-represented.

Due to linkage disequilibrium, significantly associated variants may be correlated and dependent. To properly count GWAS discoveries, we define loci iteratively using the following algorithm. For a given trait, we first rank variants with p-values < 5 × 10_-8_ from the GWAS catalog based on their p-values, from small to large. We define the first locus as a 1 million basepair window surrounding the most significant variant. We then remove all variants in the locus from the list of significant variants and repeat the above procedure to define the next locus until we exhaust all significant variants for the trait. SLE and MS have the most loci identified (159 and 155 loci respectively), while PSOAR and SS have the least (9 and 10 loci respectively) (**Figure 1A**). This disparity could be due to the number of reported studies, sample sizes of each study, heritability of the disorder. It also depends on the effect sizes of causative genetic variants. Some variants involved in certain disorders may have large effect sizes. Individuals carrying the variants will almost surely develop disease. Most other variants have moderate effect sizes, and only slightly increase the disease risk.

GWAS have found pervasively shared genetic basis among autoimmune traits (12, 13). This finding has led to great interest in jointly analyzing GWAS results from different autoimmune traits. For example, Acosta-Herrera et al. conducted the first cross-disease meta-analysis of seropositive rheumatic diseases (SSC, SLE, RA, and idiopathic inflammatory myopathies) (14). This joint analysis enabled identification of five shared immune-related loci that had not been previously associated with these individual diseases. As another example, Márquez et al. performed meta-analysis on data from CEL, RA, SSC, and T1D. This not only allowed them to identify novel genome-wide associations, but also to propose new candidate treatments through drug repositioning analysis (15).

GWAS has also helped reveal the genetic etiology of disease subtypes, which is important given the extensive clinical heterogeneity. For example, Chung et al. performed a GWAS to identify risk loci associated with anti-dsDNA autoantibody production in SLE patients (16). They observed that previously identified SLE susceptibility loci are associated with higher autoantibody production in anti-dsDNA positive SLE patients compared to anti-dsDNA negative SLE patients. This study also importantly underscores the need to identify genetic loci and non-genetic factors in autoantibody-negative SLE patients.

Despite the success of GWAS in characterizing autoimmune diseases, there are areas for further improvement. For example, it is important to identify sex-specific variants, particularly as many autoimmune diseases have a sex bias that are not fully explained by hormonal differences between males and females. For example, the incidence of SS, SLE, HT, GD, scleroderma, myasthenia gravis, PBC, and RA are female biased (17), while T1D and AS are male biased (18). There are also disorders that are not sex biased, such as UC and CD (19). Currently, most studies still pool both sexes together, with little effort to identify whether there is heterogeneity in disease susceptibility variants between female and male (20). Very few studies include chromosome X in their analysis, which is an important omission that needs to be further studied (**Figure 1B**). Inclusion and in-depth analysis of chromosome X and its relation to autoimmune diseases are especially important for sex-biased diseases, e.g., most of SLE and SS cases are females.

In addition, current GWAS studies primarily focused on samples of European ancestry, and thus lack ancestral diversity (**Figure 1C**). This is a rather unfortunate omission, as many autoimmune diseases are more prevalent in non-European populations (21). The lack of diversity hinders our understanding of the etiology of autoimmune diseases. Multi-ancestry genetic studies are in great need for further discovery and refinement of disease-associated loci (22). There have been limited multi-ancestry meta-analysis efforts for SLE, RA, CEL, SSC, and T1D. These studies have helped identify novel risk loci (15, 23–32) and improve our understanding of these autoimmune diseases (23, 26, 30, 33).

## Statistical Methods for Genetic Risk Prediction

Advances in GWAS of autoimmune diseases have helped reveal biological mechanisms underlying autoimmunity. Another application for GWAS results is to predict whether an individual is at a risk of developing a disease using his/her genotype. A polygenic risk score (PRS) aggregates many risk variants identified from GWAS to formulate a score that predicts an individual’s risk for a certain disease. If the score is high in comparison to the population of healthy individuals, the patient has a high probability of developing the disease. Identifying individuals at risk can influence clinical decisions, including frequent monitoring, early detection and/or early intervention before the disease fully develops.

**Figure 2:**
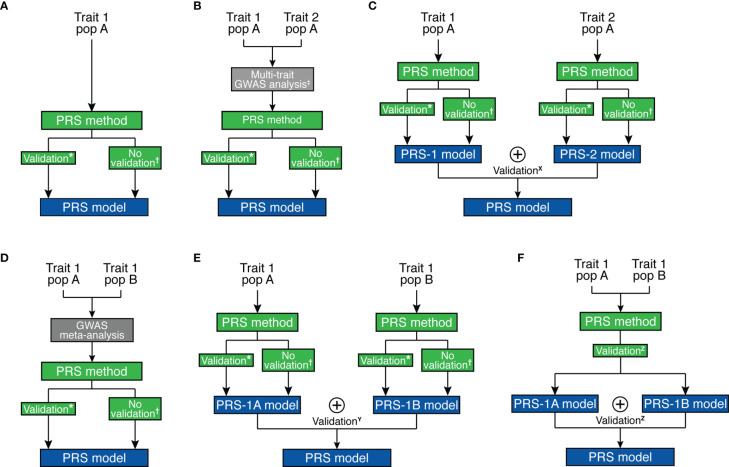
Overview of strategies for polygenic risk score model development. **(A)** Single-trait and single-ancestry framework. **(B)** Multi-trait (at GWAS level) and single-ancestry framework. **(C)** Multi-trait (at PRS model level) and single-ancestry framework. **(D)** Single-trait and multi-ancestry (at GWAS level) framework. **(E)** Single-trait and multi-ancestry (at PRS model level) framework. **(F)** Single-trait and multi-ancestry (at both levels) framework. *Pruning and Thresholding, PRSice, Pruning and Thresholding with functionally-informed LASSO shrinkage, AnnoPred, BayesR, GBLUP, JAMPRED, LDpred/LDpred2, LDpred-funct, PRS-CS, LASSOSUM. ^†^PUMAS, GCTA/SBLUP, GCTB/SBayesR, LDpred-inf, LDpred-funct-inf, PRS-CS-auto, LASSOSUM-pseudovalidation. ^‡^MTAG, wMT-GWAS, Genomic SEM. X MPS, wMT-SBLUP. Y MultiPRS, PolyPred+. Z PRS-CSx. ⊕ represents the “stacking” method to combine different risk scores.

Several methods and strategies existed for creating PRS models (**Figure 2** and **Table 1**). In general, a base GWAS summary statistic and ancestry-matched linkage disequilibrium (LD) reference panel are necessary to develop the ancestry-specific PRS model. When LD information is not available for the individuals analyzed in the GWAS, a LD reference panel from major public genomic resources [e.g. 1000 Genomes Project (61), Haplotype Reference Consortium (62)] can be used as a proxy. Some PRS methods require estimating tuning parameters, thus need an additional validation dataset (**Table 1**).

**Table 1:** A list of polygenic risk score and other relevant methods.

**Multi-trait GWAS methods**	
- MTAG (34) - wMT-GWAS (35) - Genomic SEM (36)	
**Single-ancestry PRS methods**	
*PRS methods requiring validation dataset*	*PRS methods not requiring validation dataset*
***Pruning and Thresholding*** - Pruning (37) + Thresholding (38) - PRSice (39, 40) - Pruning + Thresholding with functionally-informed LASSO shrinkage (41) ***Bayesian Framework*** - AnnoPred (42) - BayesR (43) - GBLUP (44) - JAMPred (45) - LDpred (46)/LDpred2 (47) - LDpred-funct (48) - PRS-CS (49) ***Others*** - LASSOSUM (50)	***Pruning and Thresholding*** - PUMAS (51) ***Bayesian Framework*** - GCTA (52)/SBLUP (53) - GCTB (54)/SBayesR (55) - LDpred-inf (46) - LDpred-funct-inf (48) - PRS-CS-auto (49) - SDPR (56) ***Others*** - LASSOSUM-pseudovalidation (50)
**Multi-trait PRS methods**	
- MPS (57) - wMT-SBLUP (35)	
**Multi-ancestry PRS methods**	
***Linear combination*** - MultiPRS (58) - PolyPred+ (59) ***Bayesian Framework*** - PRS-CSx (60)	

For the remaining of the section, we will review some methodological advances and challenges of the calculation of PRS for interested readers. Readers who are more interested in applications can safely ignore them and advance to the next section.

The most basic PRS method is pruning and thresholding, also known as clumping and thresholding, which involves two filtering steps. Specifically, the algorithm iteratively: 1) removes variants that are correlated with the top variant within the locus [pruning (37)] and 2) removes variants with a P-value larger than a certain threshold [thresholding (38)]. More sophisticated methods, such as LDpred (46), LDpred2 (47), BayesR (43), and PRS-CS (49) also perform shrinkage estimation by fitting the model using Bayesian methods and using a prior to model the effect size distribution of SNPs in the genome, which allows borrowing strength across different variants. More recently, AnnoPred (42) and LDpred-funct (48) methods further allow incorporation of functional priors to prioritize SNPs located within functionally-annotated regions. Another important class of methods uses penalized regression to build prediction models [e.g. LASSO regression in LASSOSUM (50)], which can be computationally more efficient than Bayesian methods.

Due to the pervasive genetic sharing between different autoimmune diseases, incorporating GWAS datasets from genetically correlated traits may improve the accuracy of genetic effect estimates, which will in turn improve the prediction accuracy of the PRS model. This is particularly appealing for autoimmune diseases with low prevalence. As it is often difficult to collect enough number of cases for less prevalent disorders, borrowing strength from other genetically-correlated autoimmune diseases is beneficial. For example, SLE is a rare autoimmune disease that is clinically and genetically known to overlap with RA and SSC (63, 64). Multi-trait PRS analysis can be performed at two different stages. First, multi-trait association methods [e.g., MTAG (34), wMT-GWAS (35), Genomic SEM (36)] can be used to improve marginal effect estimates, which we can use with other prediction methods to improve prediction accuracy (**Figure 2B**). Alternatively, “stacking” based methods create a weighted combination of PRS for different traits to enhance prediction accuracy, e.g., MPS (57), wMT-SBLUP (35). Stacking-based methods require a validation dataset to estimate weights to combine different PRS (**Figure 2C**).

Another important aspect of the PRS model is the transferability of the model across all populations. Currently, ~79% of all GWAS participants are of European descent (**Figure 1C**), which only make up for 16% of the global population. The PRS models developed for individuals of European ancestry often have reduced accuracy for prediction in non-European ancestries (65). Poor PRS transferability may be due to linkage disequilibrium differences, allele frequency differences, causal effect-size differences, and heritability differences between ancestries (59). There is great interest to develop transferable PRS integrating multi-ancestry genetic studies. There are several approaches to integrate multi-ancestry datasets for PRS prediction.

First, multi-ancestry meta-analysis of GWAS can improve marginal genetic effect estimates, which is used for a prediction model to improve prediction accuracy (**Figure 2D**). A second possible approach also uses “stacking” methods to combine PRS models [e.g., MultiPRS (58), PolyPred+ (59)] similar to multi-phenotype analysis (**Figure 2E**). Finally, multi-ancestry meta-analysis and stacking methods can both be applied [e.g., PRS-CSx (60)] (**Figure 2F**). The transferability of PRS depends on the target population and can be improved by prioritizing functional variants (66). For example, Ishigaki et al. demonstrated that the PRS performance for rheumatoid arthritis is comparable between European and East Asian populations when incorporating functional information to prioritize causal variants (67). Importantly, it still remains an open question how to best combine multi-ancestry genetic data to create a better and more transferable PRS model. Despite the advances brought by these methodologies, it is essential to enlarge non-European GWAS sample sizes. For further discussion on development, evaluation, and application of PRS, readers may refer to more thorough reviews on this topic, e.g., Chatterjee et al. (68) and Choi et al. (69).

## Availability, Accuracy and Utility of Polygenic Risk Score Models

**Figure 3:**
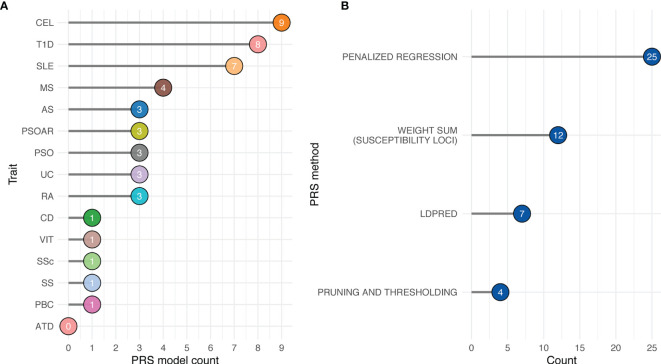
Availability of autoimmune PRS models from Polygenic Score Catalog. **(A)** Number of available PRS models by trait. **(B)** Number of available PRS models by PRS method. *Penalized regression*: LASSOSUM, snpnet, L1-penalized support vector machine. *Weighted sum (susceptibility loci)*: GWAS significant variants, HLA-specific significant variants, GWAS fine-mapped variants, and SNPs curated from literatures. *LDpred*: LDpred and LDpred2.

**Figure 4:**
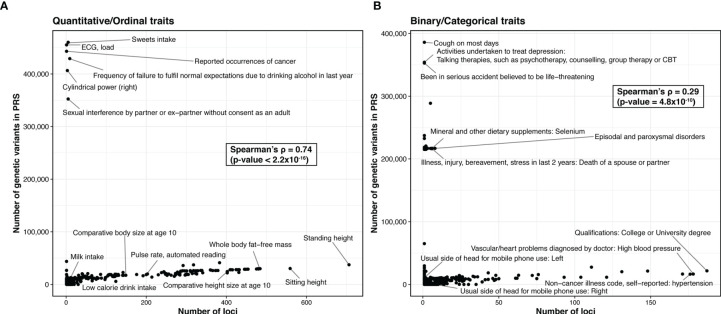
Comparison of the trait polygenicity and the PRS model size. **(A)** Quantitative/ordinal traits. **(B)** Binary/categorical traits. We apply LASSOSUM across GWAS analysis of the UK biobank data (round 2) from http://www.nealelab.is/uk-biobank/. We exclude traits that have no significant variant (p < 5×10^-8^). For binary/categorical traits, we further excluded traits with number of cases ≤5000. In total, we created polygenic risk score models for 338 quantitative/ordinal traits and 454 binary/categorical traits. We used number of loci identified in UK Biobank as a proxy for the degree of trait polygenicity.

At the time of this review, 48 PRS models have been deposited in Polygenic Score (PGS) Catalog for risk prediction for 16 autoimmune traits (**Figure 3**) (70). CEL, T1D, and SLE have the most PRS models, while to date ATD has no PRS models yet (**Figure 3A**). The most commonly used method for building the PRS model across these studies is penalized regression (50, 71–73), followed by weighted sum of the variants from established genes (e.g., from variants that reach genome-wide significance, candidate genes, etc., in contrast to scores constructed based on all variants from GWAS) (**Figure 3B**). The least used methods were pruning and thresholding (37, 38) (**Figure 3B**). Lastly, depending on the method, the number of SNPs used in the PRS model varied. LDpred2, a method assuming polygenicity, retained the most SNPs, ranging from 22,026 to as many as 566,637, while other variable selection methods used less than 2,000 SNPs in the PRS. The number of retained SNPs also critically depends on the genetic architecture of the disease. PRS of highly polygenic traits tend to contain many SNPs, while the traits that are more similar to a monogenic disorder use fewer SNPs in the PRS (**Figure 4**). Using GWAS data from UK biobank (74) along with LASSOSUM method (50), we demonstrated that the Spearman’s correlations between number of loci and number of genetic variants in polygenic risk score models are significantly and positively correlated for both quantitative/ordinal traits (**Figure 4A**; Spearman’s correlation = 0.74, p<2.2×10^-16^) and binary/categorical traits (**Figure 4B**; Spearman’s correlation = 0.29, p=4.8×10^-10^). Interestingly, a few outlier traits have many SNPs in the PRS model but relatively few GWAS loci. They are often the ones that were not extensively studied, and the sample sizes are relatively smaller. Thus, the number of known loci were relatively modest.

The most common PRS model performance metric reported is classification accuracy, as measured by the area under the curve of receiver-operating characteristic curve (ROC-AUC). Other studies report risk prediction performance as odds ratio or fold change of the proportion of cases to control in the top X^th^ percentile (e.g., top 20^th^ percentile) of the PRS distribution and compare it with the middle or bottom X^th^ percentile of the PRS distribution. Odds ratio or fold change are hard to compare between studies, as different studies use different percentile thresholds. We will only discuss PRS model performance for the studies that reported ROC-AUC.

The PRS models for T1D and CEL showed the best performance when compared to other diseases, which can be attributable to their relatively simple genetic architectures. Every PRS model of T1D had a ROC-AUC greater than 0.75, and some models had a ROC-AUC value greater than 0.9. PRS models for other autoimmune traits had moderate performance, with ROC-AUC that were greater than 0.6 but usually below 0.75. Almost all PRS models included age, sex, array type (when available), and genetic principal components as covariates in their models.

In addition to utilizing PRS for predicting disease incidence, there is also great interest in investigating the association between a high PRS and disease severity. Reid et al. observed that a high PRS for SLE was associated with earlier disease onset, increased risk of organ damage, renal dysfunction, and all-cause mortality (75). Chen et al. also observed that a high PRS for SLE correlates with poorer prognostic factors like earlier age-of-onset and lupus nephritis (76). Oram et al. observed the PRS for T1D predicted progression to insulin deficiency in diabetic young adults (77). These studies validate the clinical utility of PRS to identify individuals with high risk and susceptible to poor outcomes.

The performance of the PRS models should be interpreted with caution. Most of the PRS models were developed and evaluated using data from European ancestry populations. Due to this bias, several studies have reported decreased predictive performance when applying PRS models from European ancestry to other ancestries. Wang et al. conducted a GWAS for SLE using the Chinese population with a sample size that matches the levels of European studies (78). They developed Chinese and European specific PRS models, and these ancestry-matched models significantly outperformed ancestry-mismatched models by an average ROC-AUC of 0.14. Similarly, a PRS for T1D developed using a European ancestry population performed comparably in non-Hispanic European and Hispanic ancestries (ROC-AUC 0.86 and 0.90 respectively), but it did not perform as well in African Americans (ROC-AUC 0.75) (79). Following this observation, Onengut-Gumuscu et al. conducted a GWAS for T1D on African-ancestry participants and an African-specific PRS model improved prediction (ROC-AUC 0.87) compared to a European-based PRS model (80). Privé et al. investigated the portability of PRS models for 245 traits developed using individuals from Northwestern European ancestry in 9 different ancestry groups (72). Their analysis included several autoimmune traits: hypothyroidism, T1D, MS, UC, CD, SLE, and PSO. They observed an overall significant reduction in the accuracy of PRS models when applied to individuals from other ancestries and the performance systematically decreased as the ancestries became genetically distant from the training data used to train PRS models. Furthermore, some studies had a small number of cases in the external validation dataset (less than 100 samples). Performance metrics like ROC-AUC could be unreliable when there is a substantial imbalance between cases and controls.

## Future Directions

GWAS to date have identified numerous loci associated with different autoimmune diseases, most of which have small effect sizes. PRS enabled by large GWAS have provided an essential tool for early diagnosis and risk prediction. However, PRS only accounts for a portion of the genetic contribution, and does not fully capture other demographic, lifestyle, environmental, and clinical risk factors that may influence disease risk over time.

Besides PRS, it is also important to incorporate other clinical and demographic variables in the prediction models. For example, many autoimmune diseases have different prevalence between sexes, age group, and ancestries (81): CD and UC affect men and women equally, while SS, SLE, GD, HT, RA, and MS have a greater incidence in female (17). CD and UC have a high incidence in Caucasians and Hispanics (82), while GD is more frequent in the Asian population and less in Sub-Saharan Africans (83). Lifestyle and environmental features also modulate autoimmune disease risk. For instance, cigarette smoking is associated with increased risk of developing GD (84), SLE (85), RA (86), CD (87), and AS (88), but has shown to be associated with reduced risk of SS (89), UC (90), and CEL (91). Other factors like alcohol consumption and exercise habits also play an important role in the risk of developing autoimmune disorders (92). Some of these data are included in electronic health records (EHRs) that are now being adopted worldwide. EHRs are also a valuable source of patient history and clinical data, especially measurements for biological features that are associated with over disease onset. Physical measurements like blood pressure or body mass index, or serological measurements of antibodies or protein biomarkers provide a set of complementary information that we can use to predict the risk of disease development in addition to genetics. We believe integration of these factors with PRS could provide further improvement in estimation of disease risk.

Although limited, efforts are already underway to integrate clinical risk factors with PRS. Knevel et al. developed genetic probability tool (G-PROB) to calculate the genetic-probability (G-probabilities) of multiple related inflammatory arthritis-causing conditions (rheumatoid arthritis, systemic lupus erythematosus, spondyloarthropathy, psoriatic arthritis, and gout) in patients with unexplained joint swelling, as these patients are often misdiagnosed (10). By jointly analyzing probabilities from all diseases, their method was able to attain a reasonable diagnostic accuracy with ROC-AUC of 0.84. They further observed 35% of the patients were misclassified at the initial visit. In comparison, in 53% of patients, the disease with the highest G-probability corresponded to the final diagnosis. In 77% of patients, the final diagnosis was within the top two diseases with highest G-probabilities. This demonstrated that integration of their method with clinical information could significantly improve differential diagnosis.

Similarly, by combining a PRS of SSC with demographic and immunological parameters, Castillo et al. increased model performance by achieving ROC-AUC = 0.787 compared to ROC-AUC = 0.673 with PRS alone (93). Abraham et al. developed a PRS for CEL specific to high-risk individuals with HLA-DQ2.5 risk haplotypes, a marker that is sensitive but not specific (94). The targeted PRS model (ROC-AUC = 0.718) outperformed a PRS model that had been constructed to distinguish all CEL patients (ROC-AUC = 0.679). These studies demonstrate the utility of integrating additional risk factors with PRS, as it allows stratification of the population into different risk categories that will allow better and personalized clinical decision making.

Finally, we have provided a list of routine clinical biomarkers that are typically screened to help autoimmune disease diagnosis (**Table 2**). Systematic integration of PRS with routine clinical biomarkers is an important next step for PRS to become a useful clinical screening tool.

**Table 2:** A list of clinical biomarkers for each autoimmune disease.

Autoimmune disease	Clinical biomarkers
Ankylosing spondylitis	HLA-B27
Celiac disease	Anti-gliadin antibody, anti-endomysial antibody, anti-tissue transglutaminase, deamidated gliadin peptide, HLA-DQ2, HLA-DQ8
Crohn’s disease	Anti-Saccharomyces cerevisiae antibody, perinuclear antineutrophil cytoplasmic
Grave’s disease	Anti-thyroid-stimulating hormone receptor antibody, thyroid-stimulating hormone, free thyroxine, triiodothyronine, HLA-B8, HLA-DR3
Hashimoto thyroiditis	Anti-thyroglobulin, anti-thyroid peroxidase, anti-thyroid-stimulating hormone receptor antibody, anti-nuclear antibody, HLA-DR3, HLA-DR5
Multiple sclerosis	Oligoclonal IgG bands, HLA-DR2
Primary biliary cirrhosis	Anti-mitochondrial antibody, anti-nuclear antibody, alkaline phosphatase
Psoriasis vulgaris	Rheumatoid factor, anti-nuclear antibody, HLA-B17, HLA-C06
Psoriatic arthritis	HLA-B27
Rheumatoid arthritis	Rheumatoid factor, anti-cyclic citrullinated peptide antibody, HLA-DR4
Sjögren’s syndrome	Anti-Ro/SSA antibody, anti-La/SSB antibody, rheumatoid factor, anti-nuclear antibody
Systemic lupus erythematosus	Anti-nuclear antibody, anti-dsDNA antibody, anti-Smith antibody, anti-phospholipid antibodies, C3, C4, HLA-DR2, HLA-DR3
Systemic sclerosis	Anti-nuclear antibody, anti-centromere antibody, anti-topoisomerase I antibody
Type 1 diabetes	Islet autoantibodies, anti-glutamic acid decarboxylase, HLA-DR3, HLA-DR4
Ulcerative colitis	Anti-Saccharomyces cerevisiae antibody, perinuclear antineutrophil cytoplasmic
Vitiligo	Anti-thyroperoxidase antibody, anti-thyroglobulin antibody

## Author Contributions

CK, HM, and DL wrote the first draft of the manuscript. LC, NO, and BJ wrote sections of the manuscript. All authors contributed to manuscript revision, read, and approved the submitted version.

## Funding

This work was supported by the National Institutes of Health grants R56HG011035, R01GM126479, R21AI160138, R03OD032630, T32GM118294, T32LM012415, and U01AR071077. This work was also funded by Lupus Research Alliance and CURE funds from the Pennsylvania Department of Health. This work was also funded in part by generous support from Robert and Sevia Finkelstein. The funders were not involved in the study design, collection, analysis, interpretation of data, the writing of this article or the decision to submit it for publication.

## Conflict of Interest

The authors declare that the research was conducted in the absence of any commercial or financial relationships that could be construed as a potential conflict of interest.

## Publisher’s Note

All claims expressed in this article are solely those of the authors and do not necessarily represent those of their affiliated organizations, or those of the publisher, the editors and the reviewers. Any product that may be evaluated in this article, or claim that may be made by its manufacturer, is not guaranteed or endorsed by the publisher.
